# NeuronDepot: keeping your colleagues in sync by combining modern cloud storage services, the local file system, and simple web applications

**DOI:** 10.3389/fninf.2014.00055

**Published:** 2014-06-12

**Authors:** Philipp L. Rautenberg, Ajayrama Kumaraswamy, Alvaro Tejero-Cantero, Christoph Doblander, Mohammad R. Norouzian, Kazuki Kai, Hans-Arno Jacobsen, Hiroyuki Ai, Thomas Wachtler, Hidetoshi Ikeno

**Affiliations:** ^1^Department of Biology II, G-Node, Ludwig-Maximilians-Universität MünchenPlanegg-Martinsried, Germany; ^2^Department for Innovations, Max Planck Digital LibraryMünchen, Germany; ^3^MRC ANU, Department of Pharmacology, University of OxfordOxford, UK; ^4^Department of Informatics, Technische Universität MünchenMünchen, Germany; ^5^Department of Earth System Science, Fukuoka UniversityFukuoka, Japan; ^6^School of Human Science and Environment, University of HyogoHyogo, Japan

**Keywords:** morphology, electrophysiology, imaging, data management, neuroinformatics, cloud services, research data management

## Abstract

Neuroscience today deals with a “data deluge” derived from the availability of high-throughput sensors of brain structure and brain activity, and increased computational resources for detailed simulations with complex output. We report here (1) a novel approach to data sharing between collaborating scientists that brings together file system tools and cloud technologies, (2) a service implementing this approach, called NeuronDepot, and (3) an example application of the service to a complex use case in the neurosciences. The main drivers for our approach are to facilitate collaborations with a transparent, automated data flow that shields scientists from having to learn new tools or data structuring paradigms. Using NeuronDepot is simple: one-time data assignment from the originator and cloud based syncing—thus making experimental and modeling data available across the collaboration with minimum overhead. Since data sharing is cloud based, our approach opens up the possibility of using new software developments and hardware scalabitliy which are associated with elastic cloud computing. We provide an implementation that relies on existing synchronization services and is usable from all devices via a reactive web interface. We are motivating our solution by solving the practical problems of the GinJang project, a collaboration of three universities across eight time zones with a complex workflow encompassing data from electrophysiological recordings, imaging, morphological reconstructions, and simulations.

## 1. Introduction

Science today deals with a “data deluge” caused by the widespread use of high-throughput sensors in experiments, and the ever more complex simulations afforded by increased computational power (Moore, 1965). Both measured and simulated data need to be stored in raw form, preprocessed, contextualized with metadata, organized to facilitate queries, and then analyzed to produce scientific statements. Ideally, peer-reviewed data should also be available for replication and re-analysis to test new hypotheses as knowledge progresses.

In addition, the need for multi-university collaboration is particularly acute in neuroscience being a multilevel discipline. It tackles questions spanning disparate levels of organization such as genes, neurons, circuits, and behavior with a variety of methods including sequencing, electrophysiology, and computer simulations (Shepherd et al., 1998). Projects with such multi-university collaborations benefit from well organized coordination of the participating specialists (Cummings and Kiesler, 2007).

One challenging aspect of project workflows might concern immediate sharing of highly structured and voluminous data across labs. Tasks of such a project workflow can interdepend: a further step of the local work depends on another operation that is remotely carried out. In this case, scientific workflows allow to optimize and then more efficiently execute scientific processes (Ludäscher et al., 2009). For example, analysis results can motivate further collection of experimental data, whereupon it is clearly of advantage that they are made available once they are produced.

Proposals to alleviate the data management overhead frequently might require scientists to change and diminish their local processing workflow in order to be able to offer distributed access for collaborators to participate in the project. We propose here a novel approach which integrates seamlessly the widespread filesystem-based acquisition, analysis, and publication workflows by leveraging proven cloud synchronization technology. Our implementation of this approach, a service called NeuronDepot, enables researchers to continue interacting with the scientific project data through the filesystem and at the same time opens up the data for further processes in cloud-based web applications. In this way NeuronDepot exploits the existing substantial investment in development, acquisition, and training in local applications with their mature and rich interfaces and local access to data. We illustrate the approach with a deployment of NeuronDepot tailored to the specific needs of the GinJang project (http://projects.g-node.org/ginjang/), a complex use case that combines data from electrophysiological recordings, imaging, morphological reconstructions, and simulations.

Several initiatives have established databases to make neuromorphological or neurophysiological research data publicly available. NeuroMorpho (http://neuromorpho.org/) is a curated inventory of digitally reconstructed neurons. The goal of the project is to provide dense coverage of available reconstruction data for the neuroscience community (Ascoli et al., 2007). The neurodatabase.org project (http://neurodatabase.org) and the Collaborative Research in Computational Neuroscience (CRCNS) site (http://crcns.org) host electrophysiological data that have been specifically selected by contributing labs for the purpose of making the data available to the public. Typically, data in these databases are from studies that have been published and are provided for use in further investigations after they have served their primary purpose. Only a few projects have been designed to support data sharing in collaborative research.

The CARMEN portal (https://portal.carmen.org.uk/) allows neuroscientists to share data and programs from neurophysiological experiments. Data analysis functions are provided as services that can be applied to the data stored in the system (Austin et al., 2011). Data, metadata, and analysis workflows are accessible via a web interface.

The German Neuroinformatics Node (G-Node) provides a platform for management and sharing of neurophysiological data (http://www.g-node.org/data). Users can upload, organize, and annotate data, and make them accessible to other users or the public. Data annotation follows a flexible schema (Grewe et al., 2011) so that any metadata necessary can be entered. An API provides fine-grained data access through common languages like Python or Matlab, enabling data management and collaborative data sharing directly from the scientists' local data workflow environments (Sobolev et al., 2014a,b).

Recently, the International Neuroinformatics Coordinating Facility (INCF) established the INCF Dataspace (http://incf.org/dataspace), a cloud-based file system to share all kinds of neuroscience data.

One of the first databases integrating results from various fields like morphology, physiology, and immunohistochemistry is the Bombyx Neuron Database for assembling and sharing experimental and analytical data (Kazawa et al., 2008). Its integrative approach inspired also the development of NeuronDepot.

## 2. Scope of the NeuronDepot approach

In contrast to some of the infrastructure solutions presented above, NeuronDepot does not focus on a particular field or type of data but leaves the specifics of each data type to the well-established working environments of the participating members. NeuronDepot supports the scientist by providing a service that integrates data flows with the corresponding management and data analysis.

Beyond facilitating collaboration, the development of a database to properly store and backup all the data of the project makes it accessible to further projects. Putting data into structured databases facilitates its reuse and enables replication and verification of analyses.

### 2.1. The GinJang project and its workflow

NeuronDepot was developed around the German–Japanese collaboration GinJang (http://projects.g-node.org/ginjang/). This project provides a perfect opportunity for use-case-driven development and field-testing of the NeuronDepot infrastructure because (1) it involves three universities with several labs across multiple time zones, (2) it deals with different types of data from neuroanatomy and electrophysiology, and (3) it requires quick synchronization and reliable transfer of large quantities of raw data with complex associated metadata, including both recorded data and simulation results.

The GinJang project studies the processing of auditory signals in the honeybee. Honeybees communicate the direction and distance to food sources with hive-mates by waggle dance (Frisch, 1967). The hive-mates detect and process airborne vibration caused by the bee's wingbeat during the waggle dance, which consists of vibration pulses with a highly specific temporal pattern. Several critical interneurons for processing the airborne vibration have been identified (Ai et al., 2007, 2009; Ai, 2010; Ai and Itoh, 2012; Ai and Hagio, 2013). However, the neural processing of these vibration signals has rarely been studied: types and roles of neurons involved, their circuitry, and their development are largely unknown.

Members of the GinJang project also developed a program (SIGEN, see Minemoto et al., 2009) that is used to automatically extract and segment the morphology of interneurons that are involved in vibration processing. Goal of the GinJang project is to clarify the morphological characteristics of the vibration-processing neurons and their morphological development according to age and experience of the bees.

**Figure 1:**
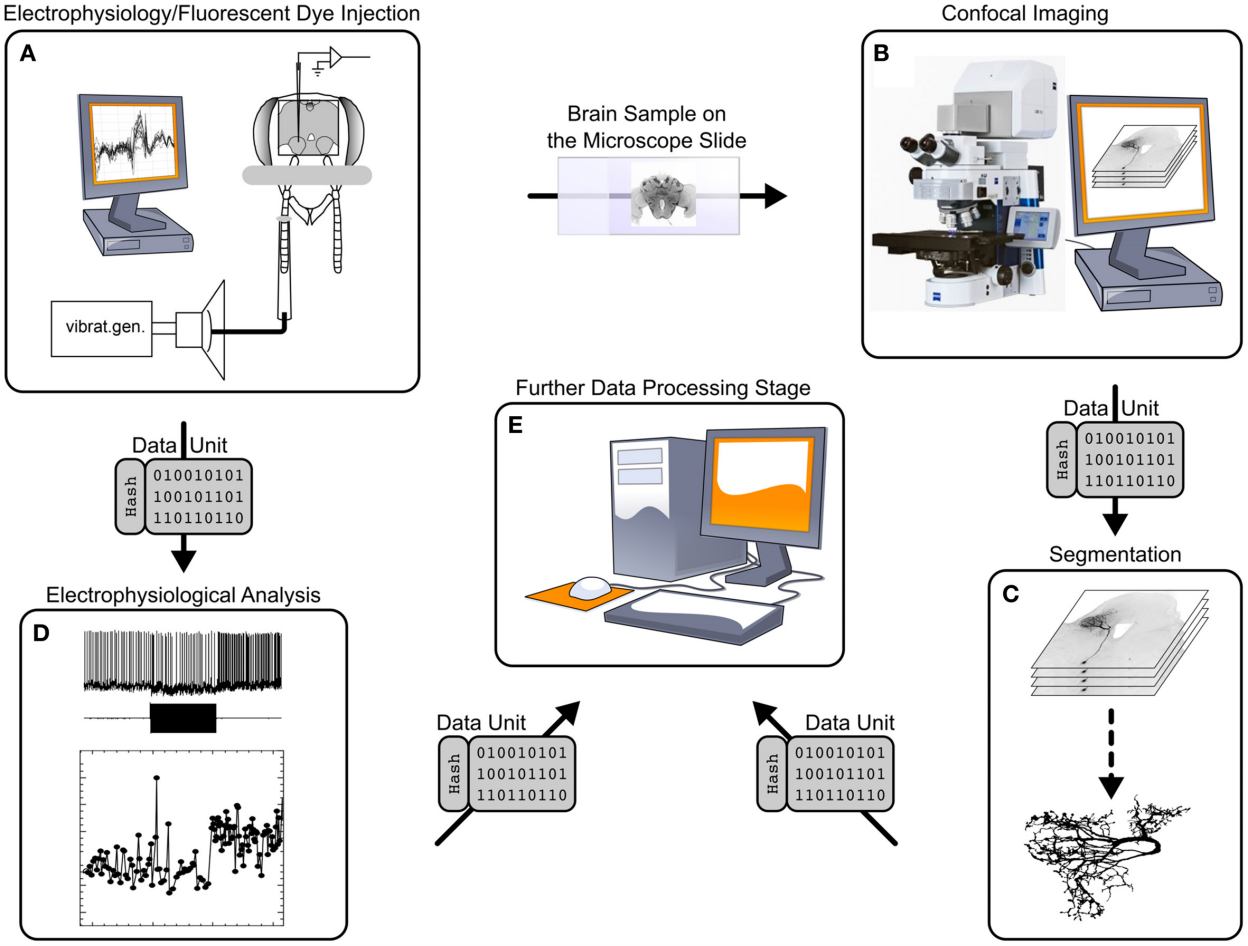
**Processing stages and data transitions of a typical workflow like the GinJang honeybee project**. (1) Processing stages **(A)** Single cell recording at a electrophysiological setup. Here, the electrical cell activity is measured at the dendrite as well as a dye is injected into the cell. **(B)** Using the brain from experiments, image stacks are created applying confocal microscope technology. **(C)** The application SIGEN computes from confocal image stacks segmentations representing the underlying neuron. **(D)** Electrophysiological recordings are analyzed with specialized software. This stage represents an entire electrophysiological infrastructure using local computers at the experimental lab but also remote G-Node-services. For simplicity, this illustration exemplary shows the result of a spike detection algorithm that identifies spikes of three neuronal units. **(E)** Further process stages follow that build upon already processed data. (2) Traditional data transition (**A** → **B**) The honeybee brain is physically moved from electrophysiological setup to the confocal microscope setup. (**B** → **C**; **C** → **E**) data units (single file, or set of files that represents a logical unit like all files of an image stack) are transferred by common tools like USB-sticks, external hard drives, Dropbox, or simply as email attachments. The same tools are applied for (**A** → **D**; **D** → **E**) but moreover dedicated web techniques for the domain of electrophysiological provided by G-Node can be applied.

**Figure 2:**
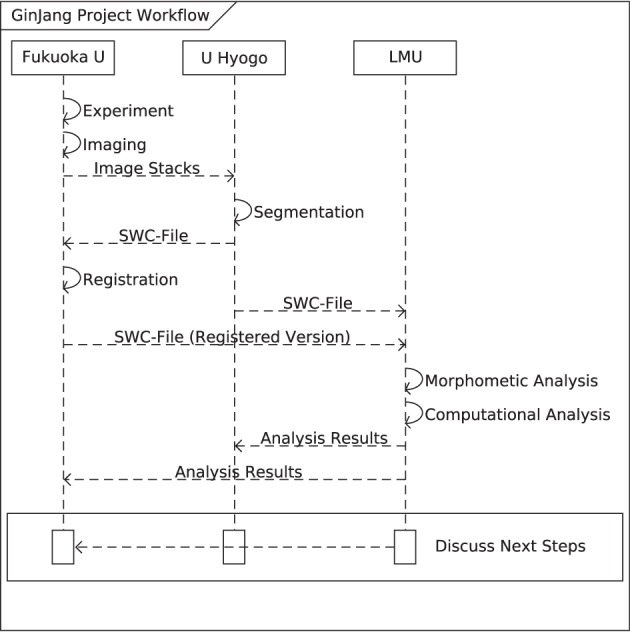
**Sequence Diagram of the GinJang workflow (morphological scope)**. The GinJang workflow starts at the Fukuoka University with two processing stages (indicated by solid arrows): the experimental data collection and the imaging processing stage. Anatomical image stacks are transferred (dashed arrow) to University of Hyogo where they are segmented. Segmented neurons are transferred to the LMU and also back to Fukuoka University where they are registered to the honeybee standard brain. Unregistered segmentation and registered segmentation are used for simulations and analysis at the LMU. Using analysis results, scientists in Fukuoka can tweak existing experiments or design new ones.

The workflow of the GinJang project is illustrated in Figures 1, 2. The experimental setup is at Fukuoka University where electrophysiological measurements (Figure 1A), electrophysiological analyses (Figure 1D), and imaging (Figure 1B) are performed. The image stacks are used at the University of Hyogo for neuronal segmentation (Figure 1C). The resulting 3D neuronal segmentations are then normalized by registering them to the Honeybee standard brain (HSB; http://www.neurobiologie.fu-berlin.de/BeeBrain/Project.html), which is done at Fukuoka University. Finally, morphological analyses, simulations, and further analyses are done at Ludwig-Maximilians-Universität München (LMU) (Figure 1E).

#### 2.1.1. Data acquisition

The vibration-sensitive neurons in the honeybee auditory system are electrophysiologically and anatomically characterized at Fukuoka University. Using sharp electrodes, voltage traces are recorded from interneurons in response to several sensory input protocols (Ai et al., 2009; Ai and Itoh, 2012; Kai et al., 2013). Then the neuron is filled with a dye and imaged at a different setup using confocal microscopy to generate anatomical image stacks (Ai, 2010; Ai and Hagio, 2013). Thus, every experiment generates three kinds of data:

- Electrophysiological data (e.g., voltage and current traces).
- Microscopy image stacks.
- Honeybee metadata (e.g., age or colony) and neuron metadata (e.g., phenotype).

#### 2.1.2. Segmentation

Image stacks are transferred to the University of Hyogo, Japan. Here, using automated image analysis software SIGEN (Yamasaki et al., 2006; Minemoto et al., 2009) the 3D structure of the neuron is extracted and stored using the SWC file format (http://www.neuronland.org/NLMorphologyConverter/MorphologyFormats/SWC/Spec.html). At this stage two kinds of data are generated:

- Segmented neuron (e.g., SWC file).
- Parameters used for segmentation (which constitute segmentation metadata).

#### 2.1.3. Registration

The morphological segmentations of the neurons are transferred back to Fukuoka University for registration into the Honeybee Standard Brain using various transformations. We use the honeybee standard brain to analyze the spatial relationships among morphologically and physiologically characterized vibration-sensitive neurons. The neuronal profiles of stained interneurons, obtained from different preparations, are segmented as explained in the previous section. Subsequently, the neuropilar outlines are traced semi-automatically with Amira 4.1 (Evers et al., 2005) and ITK-SNAP (http://www.itksnap.org/). These neuropilar label fields are used to register the segmented neuron of each preparation into the honeybee standard brain following the method described by Brandt et al. (2005). Data generated at this stage are:

- Registered neuron morphology.
- Parameters used for registration.

#### 2.1.4. Analysis

These segmentations (both registered and unregistered) are transferred to the LMU, Germany, where 3D segmentations are used for morphometric analysis and simulation studies. Multiple kinds of data are generated at this stage:

- Model files for simulations.
- Simulation metadata, e.g., parameters of simulation, location of stimulation (input) and measurements (output).
- Simulation results: visualizations and summary data.
- Morphometric analysis metadata, e.g., subregion of analysis, metrics used.
- Results of morphometric analysis: volume, surface area, number of branch points.

#### 2.1.5. Traditional data transfer methods

The workflow of the GinJang project requires multiple data transfers between diverse processing stages. These transfers were previously done via e-mail, usb-sticks, external hard drives, ftp-servers, or cloud storage services (like Dropbox, http://www.dropbox.com/). NeuronDepot replaces these traditional data transfer methods.

## 3. Requirements analysis

We asked the members of the GinJang Project to specify the features that they expect to have in NeuronDepot. Based on those we came up with the following set of requirements:

1. Data Management
  - Replacement of “manual” data transitions that are using memory. devices like e-mail, USB-sticks, external hard drives, FTP-servers, or cloud storage services.
  - Ease of metadata assignment for various kinds of data like image stacks, voltage trace, and neuronal reconstructions.
  - Interrelate various kinds of data.
  - Visibility of the current state of the project through a web browser.
  - Automatic update and synchronization across project workstations.
2. Integration
  - Maintenance of the well-established work environments of the participating scientists.
  - Minimization of the integration effort.
3. Data Security
  - Reliability of upload and download of large data.
  - Access control.
4. Automated Backup
5. Additional Requirements
  - Easy adaptation to new data-specific requirements that emerge during the project.
  - Support for automated data processing like metadata extraction, analysis, and simulation.
  - Quick overview of contents and metadata.
  - Flexible search of data.

## 4. Concept

### 4.1. NeuronDepot as a service

NeuronDepot is designed as a service. As opposed to a product, functionalities of a service are set up to meet a specific set of requirements at a point in time (Truex et al., 1999; Bennett et al., 2000; Bullinger et al., 2003). Therefore, the specific form of NeuronDepot changes as the project progresses and its requirements continually evolve. Moreover, NeuronDepot brings together other already existing service-modules, which are reassembled and configured to meet the current requirements. While building up NeuronDepot from its sub-services, we make sure that NeuronDepot stays functional even when its sub-services develop with time. Also, these developments can be utilized in evolving NeuronDepot. By offering new functionality in a way that is compatible with existing services, tools, training, and working environments, the costs of data sharing in a collaboration are brought down to a minimum while the accessibility of research assets is future-proofed.

### 4.2. Core idea

When handling a large amount of data, it is common for scientists to arrange the corresponding files in a directory tree. By doing this, they often encode metadata in the name of directories, for example, the date of recordings or experimental parameters. NeuronDepot also uses this well established principle. The difference is that NeuronDepot automatizes this. It employs a set of rules to automatically create such a directory structure and arrange the data. It uses the associated metadata (available in the database) for naming the directories. The rules for forming this directory structure can be changed. Thus, the same data can be organized in different structures as required by the scientist.

### 4.3. Definitions

In this section we define terminologies which are used in explaining NeuronDepot.

#### 4.3.1. Data unit

**Figure 3:**
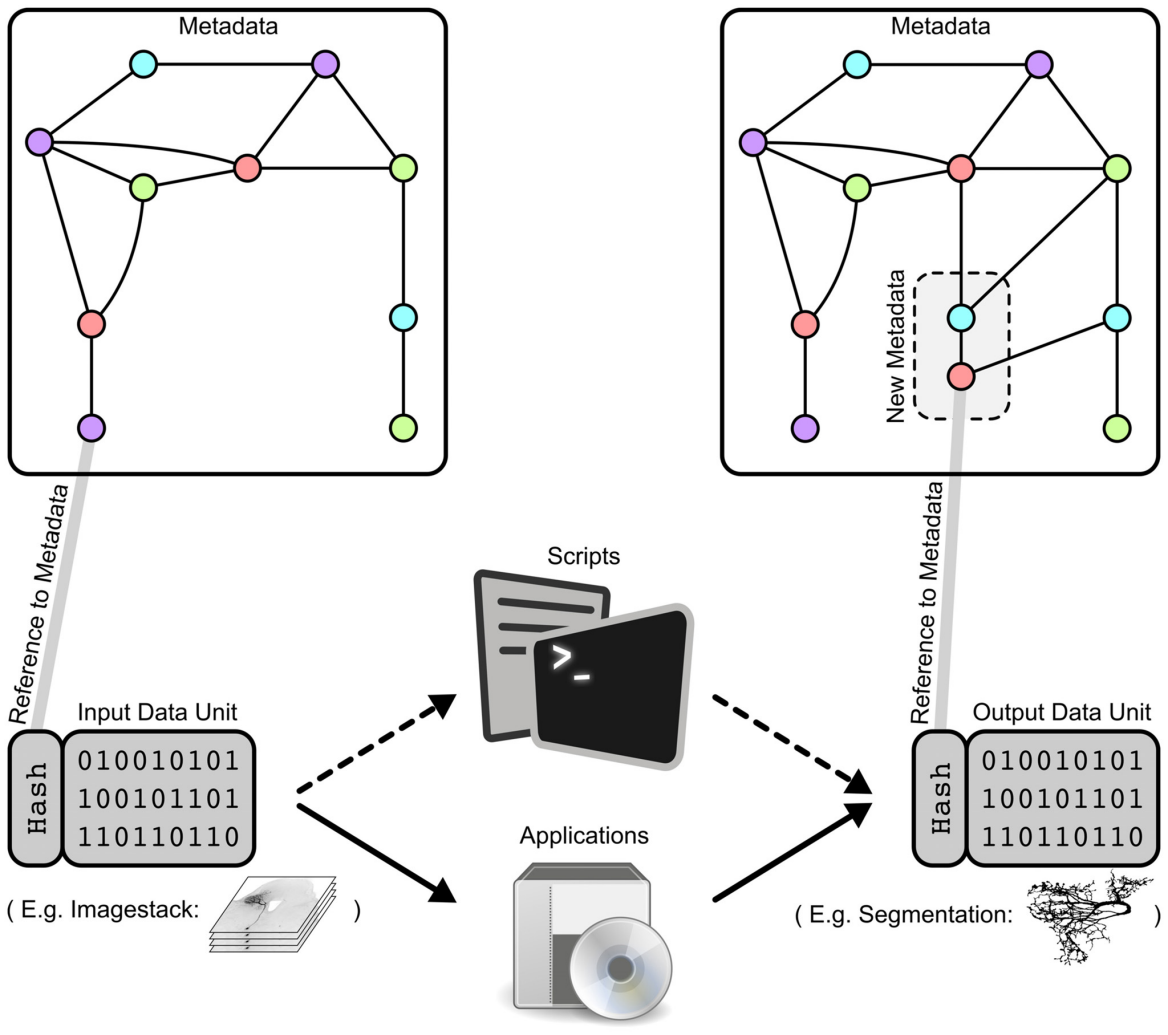
**Data units as smallest logical entity for specific data processing attached to the metadata of the project**. (Left) A data unit is connected to metadata by its unique hash value id. Metadata are illustrated here as a graph where each point represents an attribute like AGE=15, DATE=130525, or HONEYBEE=HB123. The data unit could express an image stack or a compartmental reconstruction of a specific neuron. (Middle) Data processing by a script or an applications that operates on specific input data units and that generates a new output data unit. For example, SIGEN generates from input data units expressing image stacks neural segmentations as an output data unit containing an SWC-file. (Right) Processing a data unit with a specific script or application leads to an output data unit associated with new metadata that is integrated into the existing metadata graph. According to our SIGEN-example, parameters of the segmentation algorithm are stored within the metadata graph.

A data unit (Figure 3, bottom left and bottom right) is a logical grouping of one file (trivial case) or multiple files which are generated by a single process. Examples of individual data units in the GinJang context include: an image stack consisting of several image files, the morphology of a neuron represented in a single SWC-file, or several plots and tables resulting from simulations of a neuron's electrophysiological characteristics.

#### 4.3.2. Context path and context trees

**Figure 4:**
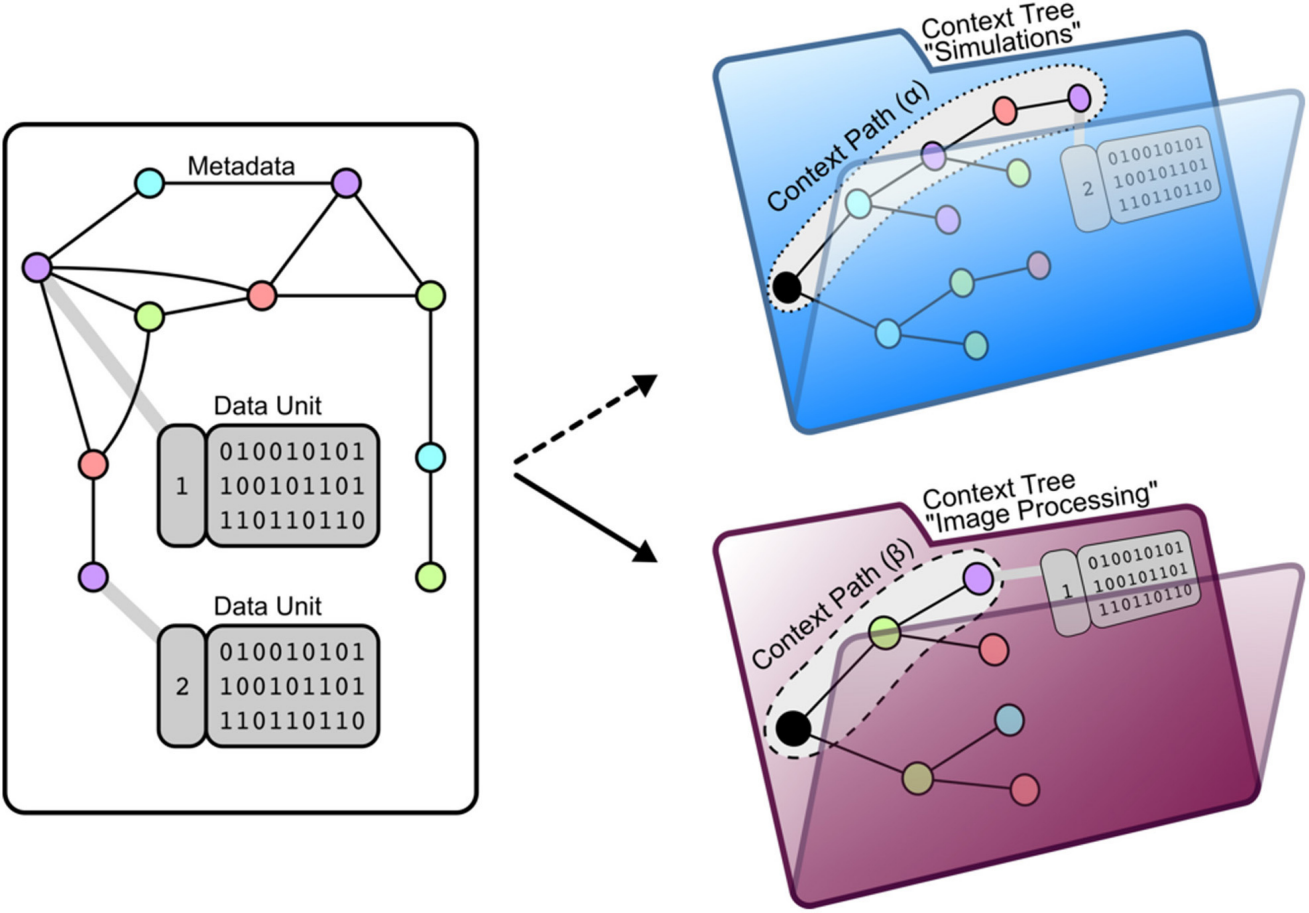
**Mapping a data unit to context trees using attached metadata**. (Left) Each data unit is connected to metadata. Using this metadata allows the organization of data units by two aspects: The entire data of a project can be (1) sub-divided into divers subsets of data where (2) data units are arranged within a tree structure where the nodes represent metadata and the leafs represent the data unit. As a meaningful specification of such a arrangement depends on the context of data usage, we call this arrangement *context tree*. (Right) One context tree of NeuronDepot arranges project data for the morphometric processing stage. The file format SWC serves as a filter argument as just SWC-files are needed for simulation. Metadata attributes LABOR_STATE, REGION, HONEYBEE_ID, and SIGEN_PARAMETERS serve for grouping. *Example of context path (α) pointing to a data unit containing a segmentation:* /forager/left_DL/HB130427/D20V05C01S01/morphology.swc Another context tree of NeuronDepot arranges project data for our imaging processing stage. Here, the project data are reduced to image stacks. Metadata attributes HONEYBEE_ID and REGION serve for grouping. *Example of context path (β) pointing to a data unit containing an image stack:* /HB130427/left-DL/^*^.tiff

Any data unit can be uniquely identified by a subset of the metadata attributes associated with it (Figure 3). We define the context path of a data unit as an ordered list of the specific attributes that uniquely identify it (Figure 4). This context path can be used to construct a path in the file system where the order of metadata attributes corresponds to the hierarchical levels in the file system.

*Example*: If a member of the project wants to analyze one particular segmentation of neuron NRN-1 of honeybee HB123, the following two paths leading to the corresponding data unit would represent these attributes:


(1) HB123/NRN-1/segmentation/
(2) segmentation/HB123/NRN-1/


The desired order of the attributes depends on how the data units are to be queried for specific analyses: the path order is projected into a hierarchy and therefore defines different grouping levels specific analyses.

#### 4.3.3. Projection

A projection is the representation of a context tree within the file system. It is comparable to materialized views of relational database management systems.

### 4.4. Design considerations

The architecture of NeuronDepot follows these principles.

#### 4.4.1. Incorporation of existing open source components

The open source ecosystem holds multiple solutions solving very specific tasks. Some examples are SQLAlchemy^1^ (for controlling the persistence of objects by mapping them to database structures), numpy ^2^ (solving highly optimized numerical tasks), or matplotlib^3^ (illustrate data by drawing graphs and figures). Moreover, the community of neuroinformatics has added several domain-specific tools for simulations, analysis, and processing of data from the field. We have incorporated some of these solution while developing NeuronDepot (see section 6). NeuronDepot is also structured so that other such solutions can be integrated to it.

#### 4.4.2. Utilization of established cloud services

Cloud services have rapidly emerged as a widely accepted paradigm built around core concepts such as on-demand computing resources, elastic scaling, elimination of up-front investment, reduction of operational expenses, and establishing a pay-per-use business model for information technology and computing services. The use of cloud services helps to reduce development time and effort.

## 5. How NeuronDepot works

### 5.1. Data arrangement: Flat on the server and hierarchical on user work stations

NeuronDepot applies systematically the principle of using folder names and file names as carrier for metadata describing the data contained in the filesystem. For flexibility, the collection of data units is stored in a central server in a flat structure where each data unit has a unique identifier, and the metadata are kept separate and referenced to those identifiers. When users define a subset of data they are interested in, along with a hierarchical arrangement that suits their needs, NeuronDepot creates a user- and task-specific context tree as a hierarchy of symbolic links with the data units at the leaves. By exposing these hierarchies to a synchronization daemon, the projection is made available to every workstation that subscribes to it.

### 5.2. Data assignment

NeuronDepot also leverages advances in synchronization technology for the data upload process: the user simply places new data units in a designated floating folder (comparable to the Camera Upload folder of Dropbox, see section 5.3). This folder is synchronized to the server. Then, the data units appear as available for metadata assignment via a graphical user interface of NeuronDepot. Once metadata assignment is complete, data units can be projected, as described above, to hierarchies that are adapted to the local users' workflows. Data units are now also accessible to cloud analytic services that directly query the metadata database without demanding a specific projection, as these clients are not constrained by the hierarchical data model of filesystems. NeuronDepot thus maintains consistency all the way from the scientists' local copy of acquired data to the cloud-based analysis platforms.

### 5.3. Cloud-based data flow

**Figure 5:**
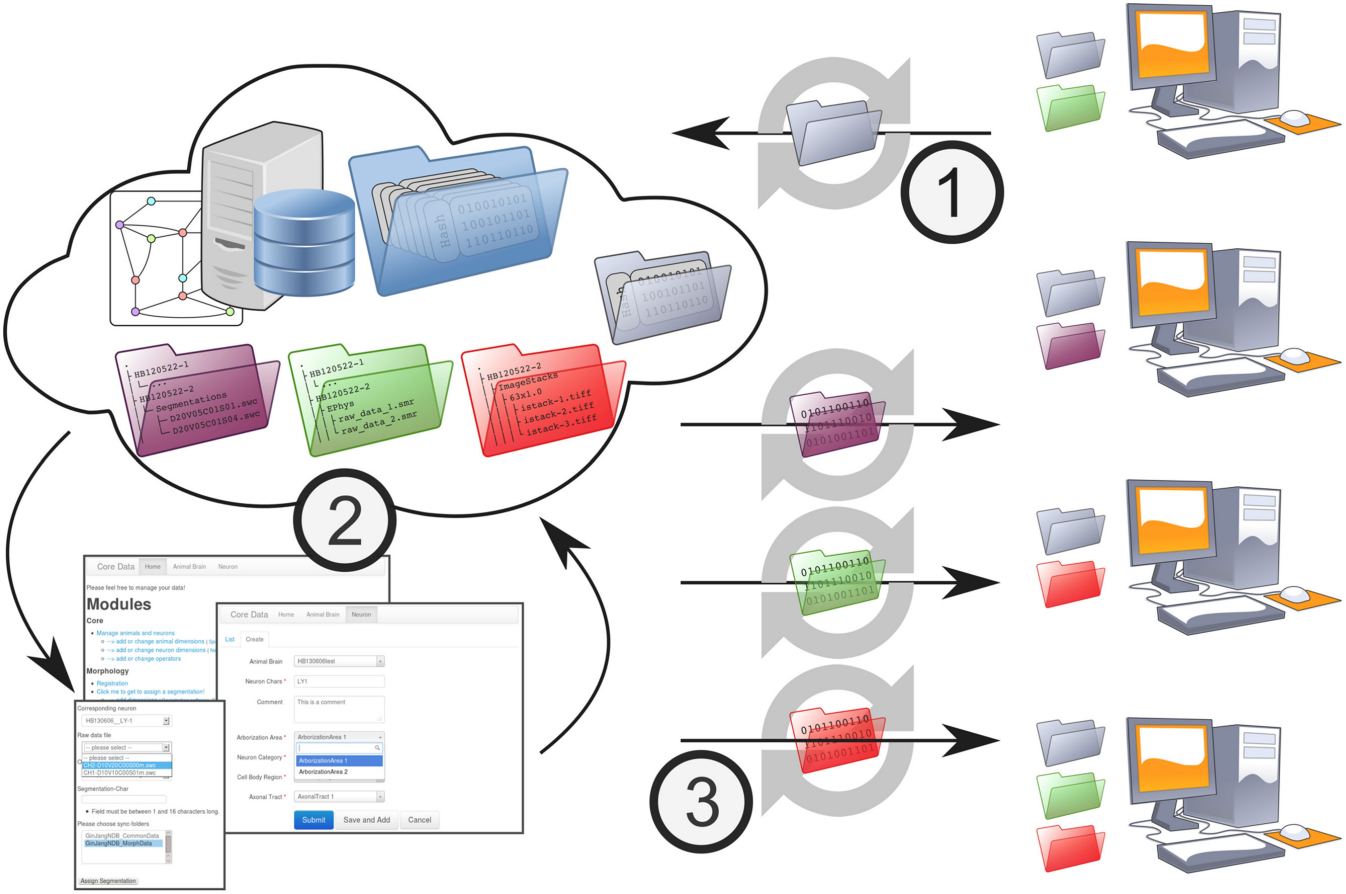
**NeuronDepot and its data flow**. NeuronDepot is based on GWDG Cloud Share and simple Flask web-apps that use modern database management systems. GWDG Cloud Share keeps all local computers that are involved in the project synchronized with the server and, therefore, synchronized among each other. This core synchronization is based on the file system. In order to integrate files into workflows, the system provides two types of base folders: floating folders for upload (gray) with read/write permissions for project members and multiple data folders (purple, green, red) with read-only permissions for project members. The data transition workflow consists of three steps: (1) new data units are stored within the floating folder and synchronized to the server. (2) Synchronized data units within the floating folder are assigned to the existing project data via a web application. As NeuronDepot's web GUI uses responsive web design it provides optimal viewing experience—easy reading and navigation with a minimum of resizing, panning, and scrolling—across a wide range of devices from mobile phones to desktop computer monitors (Marcotte, 2010). The system ensures that all data is correctly related to each other and that all data stay consistent. Project members can plug scripts into this assignment process to automate and facilitate data processing. Moreover, the system provides diverse reports to brief the scientists about the current state or about recent changes. (3) NeuronDepot distributes data units back to project members. According to the underlying context tree, NeuronDepot synchronizes projected context tree folders by cloud storage services to the workstations of the scientists.

NeuronDepot's mechanism for data transmission is based on synchronization by GWDG Cloud Share (Figure 5). This cloud storage service is used to keep all local computers that are involved in the project updated by the server and, therefore, updated among each other. This core update process is based on synchronization on the file system level. In order to integrate data units into workflows, the system provides two types of base folders: floating folders (Figure 5-1) and context tree folders (Figure 5-3). Floating folders are provided with read/write permissions for project members. Data within floating folders are not assigned to the metadata structure and, therefore, are in a floating state. Floating folders are part of the data-assignment process (Figure 5-2). The second type of folder is the context tree folder with read-only permissions for project members that synchronize projected context trees to the local work environment.

The underlying data transfer workflow replaces traditional transfer methods as described above and consists of three steps: (1) new data units are stored within the floating folder and synchronized to the server. (2) Synchronized data units within the floating folder are assigned to the existing project data via a web application. As NeuronDepot's web GUI uses responsive web design it provides optimal viewing experience—easy reading and navigation with a minimum of resizing, panning, and scrolling—across a wide range of devices from mobile phones to desktop computer monitors (Marcotte, 2010). The system ensures that all data are correctly related to each other and that all data stay consistent. Project members can plug scripts into this assignment process to automate and facilitate data processing. Moreover, the system provides diverse reports to brief the scientists about the current state, or about recent changes. (3) NeuronDepot distributes data units back to project members. According to the underlying context tree, NeuronDepot synchronizes projected context tree folders by cloud storage services to the workstations of the scientists.

## 6. System architecture

### 6.1. Graphical user interface

**Figure 6:**
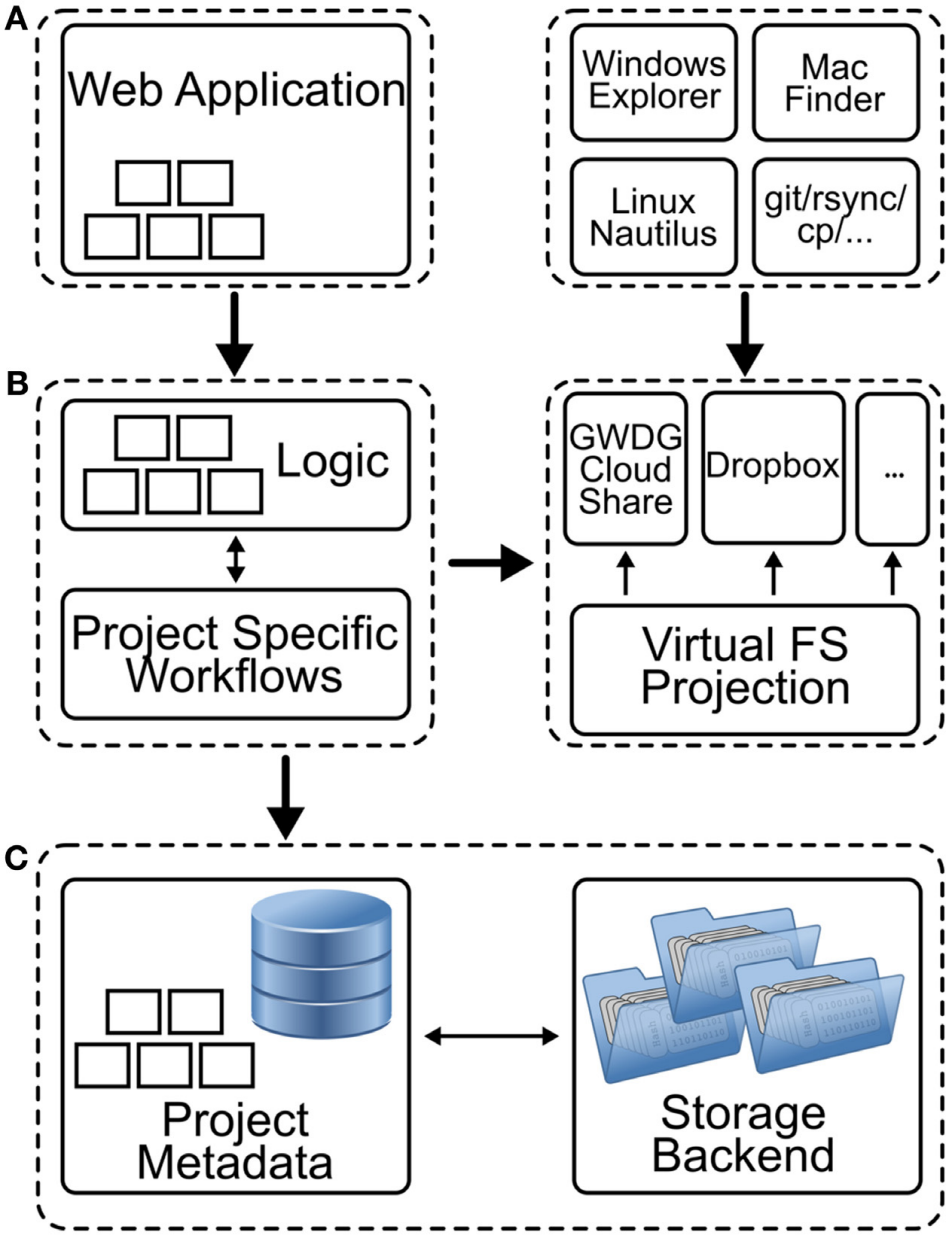
**Schematic representation of the different components of NeuronDepot. (A)** The graphical user interface consists of two parts: a web application and local applications. The web application (*left*) provides forms for assigning data, enter metadata, and annotating data with metadata. The upload and download processes are handled by GWDG Cloud Share incorporated by the filesystem projection layer (see below). Here, scientists can use established tools like Windows Explorer, Mac Finder, Linux Nautilus, or other file managers to copy files for upload in dedicated folders (*right*) which are connected to the cloud services. **(B)** The business logic (*left*) encodes the NeuronDepot logic rules that determine how data can be created, red, updated, and deleted. Moreover, when new data items are added, deleted, or modified, project-specific workflows can be triggered for each processing stage (*illustrated by five small rectangles*). A Virtual Filesystem Projection layer (right) maps data items and directories to GWDG Cloud Share synchronization clients based on project-specific metadata and provides a consistent view of the file system structure. **(C)** Within a project database additional metadata are stored. This can be metadata which was extracted by a computational workflow or manually entered data. The responsibility of the storage backend is to consistently store data items and provide abstractions for the file system projection layer.

The architecture underlying NeuronDepot consists of individual layers and components (Figure 6). Users can access NeuronDepot via a web application or through cloud storage synchronization clients. NeuronDepot distinguishes two kinds of users: registered project members which can manage the entire project data and administrators with global permissions including user management. NeuronDepot uses OpenIDs (http://openid.net/) for authentication.

The graphical user interface (GUI) consists of two parts: a web application (Figure 6A, left) and local applications (Figure 6, right). The web application provides forms for assigning data, entering metadata, annotating data with metadata, and deleting data units and metadata. We used the micro web framework Python-Flask (http://flask.pocoo.org/) for rapid development.

Upload and download processes are handled by GWDG Cloud Share (https://powerfolder.gwdg.de/) incorporated by the virtual filesystem projection layer (see below). Thus, scientists can use established tools like Windows Explorer, Mac Finder, Linux Nautilus, or other file managers to copy files for upload in dedicated folders (Figure 6A, right).

### 6.2. Business logic and virtual filesystem projection

The business logic (Figure 6B, left) encodes the NeuronDepot logic rules that determine how data can be created, read, updated, and deleted. We therefore propose a Virtual Filesystem Projection layer (Figure 6B, right) which can map data items to cloud storage synchronization clients based on project-specific metadata and provides a consistent view of the file system structure for computational workflows.

A workflow is composed out of multiple tasks. Typically tasks extract metadata, index data items, manipulate images or calculate statistics. Workflows are created in Python with the help of libraries like Snakemake (Köster and Rahmann, 2012).

Workflows are triggered explicitly by user interactions over the web frontend or implicitly by the Virtual Filesystem Projection layer when new files are added. The execution state of the workflow is displayed in the web application.

The filesystem projection layer projects the data items based on metadata to directories and files. The hierarchy of directory tree is generated by rules using project metadata and the data items which are controlled by the persistence layer.

### 6.3. Persistence layer

The persistence layer (Figure 6C) consists of the following two components:

**Project Metadata** Within a project database additional metadata are stored. This can be metadata which were extracted by a computational workflow or manually-entered data. For mapping Python objects to database objects we use SQLAlchemy (http://www.sqlalchemy.org/) storing metadata in an PostgreSQL (http://www.postgresql.org/).

**Storage Backend** The responsibility of the storage backend is to consistently store data items and provide abstractions for the file system projection layer. NeuronDepot uses Camlistore (https://camlistore.org/), which stores files like a traditional filesystem. Moreover, it's specialized in storing higher-level objects.

## 7. GinJang using NeuronDepot

In the context of the GinJang project, NeuronDepot manages image stacks and morphological reconstructions of neurons (see section 2.1). The database contains all the image stacks and neuronal reconstructions currently being analyzed as part of the project. It also contains the associated metadata (see section 2.1). The web application presents all the data annotated with metadata in easily readable tables so that the scientists can keep track of it. Such a central presentation of all the data and metadata of the project is useful during the web-based discussions of collaborators in tracking the progress of the project.

By using NeuronDepot, the process of sharing data between the collaborators has been made simple. The data are uploaded at the source of generation once and is automatically made available to the workstations where it is analyzed. Any further changes to this data, for example, if an improved neuronal reconstruction is generated, is automatically made available to the collaborator who is analyzing reconstructions. Thus, data sharing is achieved with minimum manual intervention.

The data assigned to NeuronDepot are analyzed by two collaborators (at University of Hyogo, Japan, and at LMU, Germany), each requiring them in a different hierarchical structure for their analyses. NeuronDepot automatically provides the data in the structure the collaborators specify and thus alleviates the need for manual organization.

## 8. Discussion

### 8.1. Adaptability

The system architecture of NeuronDepot can be conceptually divided into two parts: the core engine, which is not specific to any processing stage, and plain and focused modules, which are project-specific. In the GinJang project, segmentation is a processing stage that is implemented as a dedicated plain module storing, analyzing, and reporting segmentation data. Moreover, such a module provides all the required features for the data and metadata produced by this processing stage like connecting it to other existing data in NeuronDepot, handling upload of this data and specifying the information necessary while presenting it to the user.

NeuronDepot can be adapted to other projects by incorporating project-specific plain modules upon its core engine. These plain modules correspond to the different processing stages of a project, while the core engine remains the same.

### 8.2. Distinguishing features of NeuronDepot

NeuronDepot provides data via file system. This opens up a plethora of tools that are available at the local work bench like (1) desktop search using diverse indexing methods (spotlight, locate, Copernic, Google Desktop), (2) file system explorers (for searching and sorting), (3) Backup, (4) Version-Control, (5) Unix-world applications like grep, find, and tree (since “everything is a file”), (6) transmission protocols like ftp, ssh, and http, and (7) file synchronization services.

An important feature of NeuronDepot is the isolation of the upload process from the GUI. In the conventional upload process the user indicates the file to be uploaded and waits until the upload process is finished. This way of uploading can be very inconvenient when uploading large files (several hundreds of MBs). This problem is further compounded when the network connection is not stable. Our approach solves this problem by isolating the upload process from the data assignment process. The upload process of NeuronDepot consists of two steps. Data is copied into the GWDG Cloud Share and then assigned from there to the database using the GUI. This upload procedure facilitates assisted assignment of data since the data are available beforehand. Certain analysis scripts can be started on the data in the virtual file system and its results can be later used during the assignment of the data via the web-GUI.

At the end user, a subset of the data in the database is presented in a tree structure. Such a representation of a desired subset of the data in a hierarchical structure provides a partitioning/grouping of the data which becomes very handy if the user intends to perform analysis or comparison on a specific subset of the data.

In NeuronDepot, a specific subset of data is encapsulated into an entity via the concept of context trees. Such an encapsulation facilitates management operations in which treatment of the subset of the data as an entity is essential such as referencing, tagging, and sharing. This is very much like a book encapsulating a set of concepts/facts and making them a single entity.

### 8.3. Comparison with other systems

Other file-based solutions for collaborative data sharing provide access through a web browser like the web platforms of CARMEN (https://portal.carmen.org.uk/) and G-Node (http://www.g-node.org/data). There, manual download is required to access new data when a dataset has been updated, whereas in NeuronDepot the new data are automatically provided locally.

CARMEN enables access to analysis services on its platform (Austin et al., 2011). G-Node provides access to data in a common representation through an API (Sobolev et al., 2014a) with client tools for integration with the scientist's analysis scripts (Sobolev et al., 2014b). NeuronDepot complements these approaches by presenting the data in the usual file system way. This is particularly useful for collaborations between specific labs where all partners know how to access the data.

Unlike with other existing solutions, using NeuronDepot does not require learning a new GUI or any other infrastructure specific usage features since NeuronDepot provides the data as directory trees to the user. Having this feature, project members could keep their established working environments. In other words: NeuronDepot adapts for existing workflows whereas other systems require the scientist to adapt its workflow to the new system.

### 8.4. Further directions, limitations, and open questions

A package/extension for an existing web-framework like Flask or Django can be developed by reorganizing the system components of NeuronDepot. Several existing solutions of the Open Source Ecosystem were used in the development of NeuronDepot and this is a way of contributing back to it. Moreover, it serves as a good building block for the development of new data software.

As explained in section 4.1, NeuronDepot is a service which develops as the associated project progresses. In the context of the GinJang project, extensions to NeuronDepot are being developed which automate morphological analysis and simulations using the neuronal reconstructions.

At the moment, the context trees used to provide the user with data are hard-coded. The user has to communicate with the developers to have different data structures provided. This process can be slow and can prove to be a hindrance to the scientist's work. A service can be incorporated which enables the user to specify what data structure is needed. This would reduce the user's dependence on the developers and also allow the user to quickly adjust the data that are required from NeuronDepot.

## 9. Conclusion

With this software architecture, we contribute an approach to scientific data workflow and specifically a tool to the neuroscientific infrastructure. NeuronDepot's principal merit is that it integrates smoothly with established tools and resolves the transition from local to cloud-based processing. In doing so, it enables researchers to leverage the advantages of cloud services while not requiring them to relinquish control of their data or analysis.

### Conflict of interest statement

The authors declare that the research was conducted in the absence of any commercial or financial relationships that could be construed as a potential conflict of interest.

## Footnotes

^1^http://www.sqlalchemy.org/

^2^http://www.numpy.org/

^3^http://matplotlib.org/
